# Climate-Impact Uncertainty in Bio-Asphalt–Rubber Binders: Probabilistic Cradle-to-Gate Screening of Bio-Oil Inventory and Crumb Rubber Allocation

**DOI:** 10.3390/polym18172107

**Published:** 2026-08-30

**Authors:** Yemao Zhang, Xijuan Zhao

**Affiliations:** 1 School of Civil Engineering and Architecture, Nanjing Institute of Technology, Nanjing 211167, China; 2 Jiangsu Key Laboratory of Intelligent Construction and Smart Operation & Maintenance of Power Infrastructure, Nanjing 211167, China; 3 School of Management Engineering, Nanjing Institute of Technology, Nanjing 211167, China

**Keywords:** bio-asphalt–rubber binder, probabilistic life-cycle assessment, bio-oil, crumb rubber, allocation uncertainty

## Abstract

Bio-asphalt–rubber (BAR) binders combine plant-based bio-oil, end-of-life tire crumb rubber, and petroleum asphalt binder, but their climate advantage remains uncertain because bio-oil supply chains, asphalt-binder inventories, and tire-rubber allocation choices can substantially change cradle-to-gate results. This study develops a probabilistic cradle-to-gate life-cycle assessment for one metric ton of binder at plant gate. Eight alternatives were evaluated: a neat petroleum binder, a rubberized binder, a bio-oil modified binder, and five BAR binders with crumb rubber contents of 20–30% and bio-oil contents of 5–15%, expressed relative to neat asphalt mass. The model includes A1 material production, A2 inbound transport, and A3 binder blending energy. Plant-based bio-oil was represented using a literature-derived inventory, while crumb rubber was evaluated under cut-off, avoided-burden, and clinker-fuel system-expansion scenarios. A 10,000-iteration Monte Carlo simulation propagated inventory, transport, energy, and allocation uncertainty. Under cut-off allocation, mean GWP decreased from 496 kg CO2e/t for the neat binder to 446–471 kg CO2e/t for BAR binders, with BAR_30CR_15BIO showing the lowest mean impact and a 98.8% probability of outperforming the control. Avoided-burden allocation strengthened the apparent benefit, whereas system expansion against clinker fuel reversed the conclusion for rubber-containing alternatives. Results show that BAR can reduce binder-level GWP, but the conclusion is not inherent to the material; it depends strongly on tire-rubber counterfactuals and asphalt-binder inventory assumptions.

## 1. Introduction

The decarbonization of asphalt pavements increasingly requires attention to the upstream production of binders and modifiers, not only to plant operations or construction logistics [1]. In pavement life-cycle assessment (LCA), asphalt binder often dominates the material-production contribution because it carries a much higher greenhouse-gas intensity per unit mass than mineral aggregate [2,3]. The National Asphalt Pavement Association (NAPA) reported that asphalt binder manufacturing estimates in the United States span 390–578 kg CO_2_e per metric ton, and that asphalt binder is the major contributor to upstream raw-material emissions for a typical virgin asphalt mixture [4,5]. These inventory differences are not trivial methodological details: they arise from refinery allocation rules, terminal operations, crude-slate assumptions, and data boundaries [6]. Consequently, binder substitution strategies should be evaluated with explicit uncertainty rather than with a single deterministic emission factor.

Bio-based binders and extenders have been proposed as one route for reducing petroleum dependence and improving the circularity of road materials [7,8]. Bio-oils can be derived from wood fiber, waste oils, animal manure, agricultural residues, and other biomass streams through pyrolysis, liquefaction, alcoholysis, acidolysis, or biorefinery pathways [9,10]. Prior LCA work has shown that bio-modified binders can offer climate and energy advantages in some contexts, but that benefits depend on the bio-oil source, co-product treatment, processing energy, and biogenic carbon accounting [11,12]. For example, Samieadel et al. compared conventional asphalt binder with a swine-manure bio-modified binder [2], while Zhu et al. assessed plant-based bio-oil extended bituminous binders and showed that fossil GWP can slightly increase if the upstream processing burden of bio-oil is counted without a biogenic carbon credit [13]. These findings indicate that bio-oil should not be treated as a burden-free petroleum substitute.

In parallel, crumb rubber from end-of-life tires has long been used in asphalt rubber and rubberized asphalt mixtures [14,15,16]. Crumb rubber can improve elasticity, rutting resistance, fatigue resistance, and low-temperature behavior, but it can also increase binder viscosity, blending temperature, storage-stability challenges, and plant energy requirements [17,18,19]. LCA and life-cycle cost studies of rubberized asphalt have reached mixed conclusions. Some studies find that rubberized asphalt can be environmentally and economically favorable when service-life extension is sufficient [20,21]. Other studies emphasize that the allocation of end-of-life tire burdens and credits can control the conclusion, especially when the counterfactual use of tires is energy recovery in cement kilns or clinker production [22,23]. Thus, the environmental value of crumb rubber is conditional on a defined waste-management baseline.

Bio-asphalt–rubber (BAR) binders combine these two material strategies: bio-oil is used as a renewable extender, viscosity reducer, or rubber-compatibility enhancer, while crumb rubber provides polymeric elasticity and a high-value outlet for tire waste [24]. Recent binder-scale research has shown that bio-oil can reduce the viscosity penalty of rubber modification and enable lower-temperature or shorter-time preparation. Xue et al. reported that binders containing 20% crumb rubber and 5–15% bio-oil exhibited improved rheological grades relative to neat asphalt [18]. Ju et al. evaluated high-rubber-content modified bio-asphalts with 20–30% crumb rubber and 5–15% bio-oil [25]. These material results make BAR binders scientifically and industrially attractive. However, they do not by themselves establish a cradle-to-gate climate advantage because the material is a coupled system: reducing petroleum asphalt mass lowers one burden, adding processed crumb rubber and bio-oil adds others, and changing the rubber counterfactual can alter the sign of the result.

Recent studies have further advanced the environmental assessment of bio-based and recycled asphalt materials. Muscò et al. reviewed bio-based paving binders and showed that their properties and environmental implications vary substantially with biomass source, conversion technology, and degree of petroleum-binder replacement [26]. Ju et al. evaluated bio-oil and high-rubber-content modified asphalt by integrating rheological performance, economic analysis, and LCA, thereby demonstrating the technical potential of the proposed formulations [25]. Nevertheless, such formulation-level studies have generally relied on fixed inventory and waste-management assumptions and have not explicitly quantified whether their environmental conclusions remain robust under joint bio-oil and crumb-rubber uncertainty. For crumb-rubber systems, Cao et al. showed that the environmental ranking of alternative rubber-modification technologies changes with the adopted system boundary [27], while Sudan et al. demonstrated that the impacts of producing crumb rubber and other recycled tire products depend strongly on processing technology and electricity supply [28]. These findings indicate that recycled or bio-based material content alone does not guarantee a climate benefit; the result is conditional on the upstream production pathway, regional background system, and treatment of the displaced end-of-life tire route.

Methodological advances have also strengthened uncertainty analysis in pavement LCA. Li et al. developed a probabilistic and comparative framework that systematically represents different levels of data specificity and evaluates the reliability of decisions under incomplete pavement data [29]. Vargas Farias et al. further showed that uncertainty arises not only from input variability but also from methodological choices and parameter interactions, and applied multiple global sensitivity methods to pavement-management scenarios [30]. However, these studies primarily address asphalt mixtures, pavement structures, maintenance strategies, or road networks. They do not examine formulation-specific uncertainty in a coupled BAR binder system, where petroleum-asphalt substitution, bio-oil production, crumb-rubber processing, and waste-tire counterfactuals simultaneously determine the result. Moreover, existing bio-binder and rubberized-asphalt studies often treat these uncertainty sources separately, making it difficult to determine whether an apparent environmental advantage reflects the binder formulation itself or the inventory and allocation assumptions adopted in the assessment.

Against this background, the present study jointly evaluates bio-oil inventory uncertainty and crumb-rubber allocation uncertainty within a mass-normalized, binder-level probabilistic framework. Unlike recent pavement-level probabilistic LCAs, the analysis focuses on the environmental consequences of coupled modifier substitution before mixture- and pavement-performance data are available. Unlike existing bio-oil–rubber formulation studies, shared inventory parameters are propagated using common random numbers, and each alternative is evaluated using both its GWP distribution and the paired probability of outperforming the neat-binder control. The cut-off, avoided-burden, and clinker-fuel counterfactual scenarios are also evaluated within the same framework. The contribution therefore lies not merely in applying Monte Carlo simulation but in determining whether the apparent cradle-to-gate climate advantage of BAR binders is robust to formulation mass balance, bio-oil supply assumptions, crumb-rubber processing variability, and alternative waste-tire counterfactuals.

Accordingly, this study develops a probabilistic cradle-to-gate assessment of BAR binders at the plant gate. Eight binder alternatives are evaluated to quantify their GWP distributions, probability of outperforming a neat petroleum binder, contribution structures, and sensitivity to bio-oil inventory and crumb-rubber allocation assumptions. The framework is intended as an uncertainty-aware screening tool for identifying robust environmental advantages and the inventory assumptions that require further validation before regional or industrial implementation.

## 2. Methodology

### 2.1. Goal, Scope, and Declared Unit

The study follows the ISO 14040/14044 life-cycle assessment structure of goal and scope definition, inventory analysis, impact assessment, and interpretation [31,32]. Because the purpose is early-stage binder screening rather than full pavement design, the study adopts a declared unit of one metric ton of final binder at the plant gate. The system boundary (see Table 1) is cradle-to-gate and includes A1 upstream production of petroleum asphalt binder, crumb rubber, and bio-oil; A2 inbound transport of these materials to the binder-blending facility; and A3 binder blending energy. Aggregate production, asphalt-mixture production, construction, traffic use, maintenance, end-of-life, and material recovery beyond the binder plant gate are excluded by design. The boundary therefore estimates binder-level manufacturing carbon risk, not pavement-level life-cycle performance.

The impact category is 100-year global warming potential (GWP100), expressed as kg CO2e per metric ton of final binder [33]. The impact calculation is intentionally limited to climate change because the available BAR-specific LCI is strongest for energy and carbon flows. Other impact categories, including acidification, eutrophication, photochemical smog, particulate matter, water use, and toxicity, should be added when supplier-specific bio-oil and rubber-processing inventories become available.

### 2.2. Binder Alternatives and Mass Balance

Eight binder alternatives were defined to isolate the marginal effects of crumb rubber, bio-oil, and their combined use in BAR binders, as summarized in Table 2. The neat petroleum binder represents the control. RA_20CR represents a rubberized binder with crumb rubber equal to 20% of neat asphalt mass. BA_10BIO represents a bio-oil modified binder with bio-oil equal to 10% of neat asphalt mass. Five BAR binders cover 20–30% crumb rubber and 5–15% bio-oil, consistent with recent bio-oil/rubber modified binder experiments [18,25]. Because these modifier contents are defined relative to the neat asphalt mass, a mass-balance correction was applied to ensure that every alternative contains exactly one metric ton of final binder.

For an alternative with crumb-rubber ratio $p_{cr}$ and bio-oil ratio $p_{bio}$ expressed relative to the neat asphalt mass, the final-binder mass balance is given by

(1) $$m_{asphalt}=\frac{1}{\left(1+p_{cr}+p_{bio}\right)}$$

(2) $$m_{cr}=p_{cr}m_{asphalt}$$

(3) $$m_{bio}=p_{bio}m_{asphalt}$$

This correction is important for LCA interpretation. For example, BAR_20CR_10BIO contains 0.769 t of petroleum asphalt, 0.154 t of crumb rubber, and 0.077 t of bio-oil per ton of final binder; treating the 20% and 10% values as final-binder mass fractions would overstate the modifier mass and bias the comparison.

### 2.3. Life-Cycle Inventory and Uncertainty Characterization

Inventory parameters were represented by triangular distributions because the available BAR-related LCI data were mainly reported as ranges and central estimates rather than raw observations suitable for empirical distribution fitting. The low, mode, and high values can be directly traced to literature data or engineering assumptions, preserve physically meaningful bounds, and assign greater likelihood to the most plausible value. Accordingly, these distributions represent epistemic uncertainty rather than statistically verified variability [34]. Normal, lognormal, or beta distributions would require additional moment or shape parameters that were unavailable and would therefore introduce less transparent assumptions. Triangular distributions are also widely used in other relevant LCA and engineering applications under similar data-scarce conditions [6,35,36]. The triangular distribution was therefore adopted as a transparent and parsimonious approximation suitable for the present early-stage screening assessment. Common random numbers were used across alternatives for shared background parameters such as asphalt-binder GWP, grid electricity intensity, freight emission factor, and transport distances, so that comparative uncertainty reflects material design differences rather than Monte Carlo noise [37,38,39].

**Figure 1:**
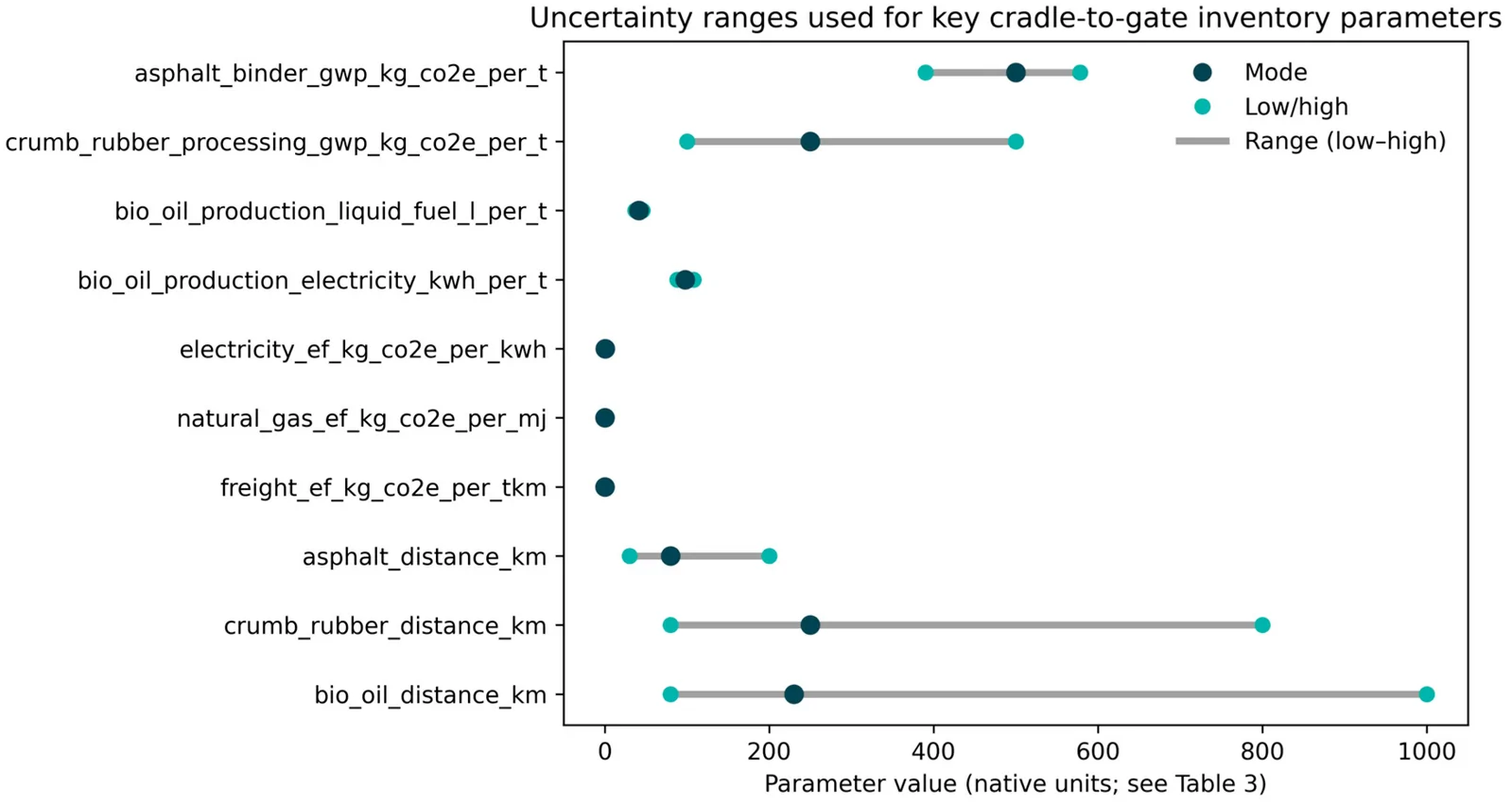
Key LCI uncertainty ranges with the low, mode, and high values used for triangular distributions.

As presented in Figure 1 and Table 3, tThe asphalt-binder GWP distribution was anchored to the range of 390–578 kg CO2e/t reported by NAPA SIP-109, which synthesizes the Mukherjee (2016) [40] and Asphalt Institute (2019) inventories [4]. A central working value of 500 kg CO2e/t was used as the mode. The crumb-rubber processing burden was treated as a cut-off processing inventory with a wide range of 100–500 kg CO2e/t to reflect uncertainty in tire collection, shredding, steel/textile separation, granulation, and powder production; the upper part of this range is supported by recent ELT recycling studies reporting rubber-powder impacts of approximately 405 kg CO2e/t [28].

The bio-oil inventory was based on a plant-based bio-oil study by Zhu et al. [13]. The bio-oil was produced from bio-based by-products of the pulp and paper industry at a Swedish biorefinery, and its production inventory included 41.3 L/t of heating oil and diesel and 97.8 kWh/t of electricity. In this study, these energy flows were converted to GWP by multiplying them by uncertain liquid-fuel and electricity emission factors. No biogenic-carbon credit was included in the reported GWP. Although the bio-oil originates from biomass, the present cradle-to-gate model does not quantify its biogenic carbon content, storage duration in the binder, or eventual release during use and end of life. Crediting biomass CO2 uptake without modeling these downstream flows could overestimate the climate benefit. The no-credit treatment was therefore adopted as a conservative screening assumption. Also, the selected bio-oil inventory should not be interpreted as statistically representative of global bio-oil production or as geographically representative of Sweden. Transparent cradle-to-gate LCI datasets for bio-oils specifically evaluated as asphalt-binder extenders remain scarce, and the selected source was one of the few studies reporting sufficiently detailed liquid-fuel and electricity inputs for probabilistic modeling. Accordingly, the inventory is used as a case-specific baseline to demonstrate a generic screening framework rather than to establish a regionalized product carbon footprint.

### 2.4. Crumb-Rubber Allocation Scenarios

Three crumb-rubber allocation scenarios were used to separate attributional screening from consequential waste-management assumptions, as listed in Table 4. In the cut-off scenario, crumb rubber enters the product system with only its collection and processing burden; no credit or penalty is assigned for the tire’s previous life or avoided alternative treatment. This is the base case because cut-off allocation is common for recycled asphalt materials in product-level inventories and avoids assigning speculative external benefits. In the avoided-burden scenario, use of crumb rubber in BAR is credited for avoiding low-value tire-management routes. In the system-expansion scenario, the counterfactual is tire-derived fuel use in clinker production; diverting tires from that route creates a foregone fuel-substitution burden. This third scenario is intentionally conservative and is motivated by studies showing that cement-kiln energy recovery can have substantial avoided GHG benefits [22,23]. It assigns a positive adjustment to crumb rubber to represent the potential foregone environmental benefit of alternative end-of-life tire uses, including energy recovery. The low, mode, and high adjustments were selected as literature-informed sensitivity levels.

The three approaches serve different decision contexts. The cut-off approach represents attributional product accounting, in which crumb rubber enters the BAR system without burdens from the previous tire life but includes the processing and transportation required for its use. This treatment is consistent with current asphalt EPD practice for secondary materials. In contrast, avoided burden and system expansion represent consequential perspectives by estimating the environmental effects of displacing alternative tire-management routes. Their results depend on the selected counterfactual and regional market conditions and are therefore treated as sensitivity scenarios rather than EPD-equivalent results.

### 2.5. Monte Carlo Model and Impact Equations

For each simulation i and alternative a, total cradle-to-gate GWP was calculated as the sum of material production, inbound transport, and binder-blending energy:

(4) $$GWP_{a,i}=GWP_{materials,a,i}+GWP_{transport,a,i}+GWP_{production,a,i}$$

Material GWP was computed as the mass-weighted sum of petroleum asphalt, effective crumb rubber, and bio-oil GWP. Transport GWP was computed by multiplying each material mass by its transport distance and the truck freight emission factor. Production GWP was computed from alternative-specific electricity use and thermal energy use multiplied by uncertain electricity and natural-gas emission factors. The effective crumb-rubber GWP was defined as the processing burden plus the allocation-scenario adjustment. A positive adjustment represents a penalty; a negative adjustment represents an avoided burden credit.

The Monte Carlo simulation used 10,000 iterations, which was confirmed to provide stable summary statistics and probability estimates through a separate convergence assessment with preliminary analysis. For each alternative, results were summarized by mean, standard deviation, median, 5th percentile, and 95th percentile. Comparative environmental preference was measured by the probability that an alternative has lower GWP than the control binder:

(5) $$P_{lower}=P\left(GWP_{alternative}<GWP_{control}\right)$$

The reduction percentage relative to the control was calculated as

(6) $$Reduction\left(\%\right)=-\frac{GWP_{alternative}-GWP_{control}}{GWP_{control}}\times 100$$

Spearman rank correlation coefficients between total GWP and uncertain inputs were calculated for each alternative to identify monotonic sensitivity patterns without assuming linear relationships.

Also, a variance-based global sensitivity analysis was conducted to complement the Spearman rank-correlation analysis and account for nonlinear and interaction effects. Sobol first-order ($S_{1}$) and total-order ($S_{T}$) indices were calculated using independent triangular input distributions and a scrambled Sobol sequence with a base sample size of 8192. The first-order index represents the variance contribution of an individual parameter, whereas the total-order index includes its main effect and all interactions with other parameters. Accordingly, $S_{T}-S_{1}$ was used to indicate the combined contribution of parameter interactions. The analysis was performed for representative binder formulations under cut-off allocation and the illustrative allocation-penalty sensitivity. Bootstrap resampling with 200 replicates was used to derive 95% confidence intervals.

## 3. Results and Discussion

### 3.1. Cradle-to-Gate GWP Distribution Under Cut-Off Crumb-Rubber Allocation

**Figure 2:**
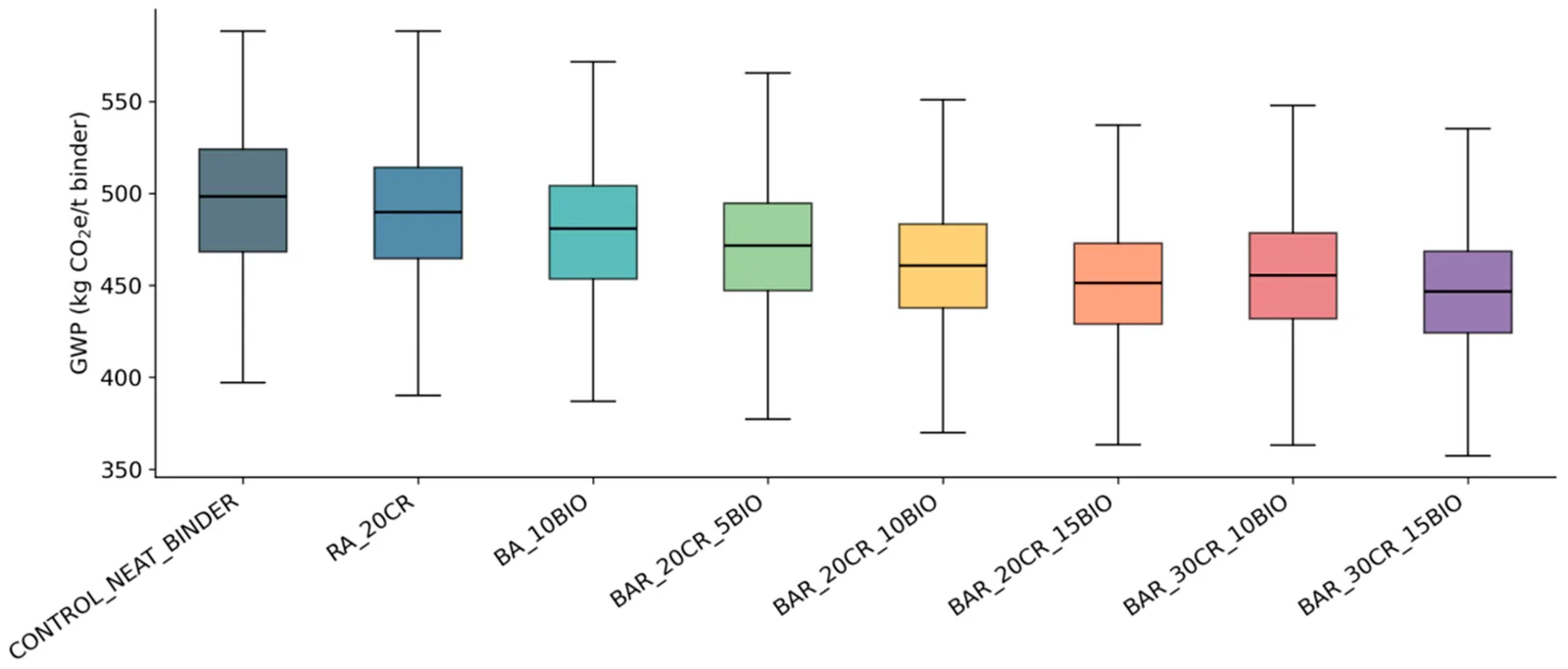
Probabilistic cradle-to-gate GWP distributions under cut-off crumb-rubber allocation. Each boxplot represents 10,000 Monte Carlo simulations for one binder alternative.

As presented in Table 5 and Figure 2, under cut-off allocation, the neat petroleum binder had a mean GWP of 495.9 kg CO2e/t binder and a fifth–95th percentile range of 428.4–558.8 kg CO2e/t. All BAR alternatives had lower mean GWP than the control, with mean values ranging from 470.8 kg CO2e/t for BAR_20CR_5BIO to 446.2 kg CO2e/t for BAR_30CR_15BIO. The reduction is driven primarily by displacement of petroleum asphalt binder, which has the highest unit GWP in the inventory. The sequence of mean GWP values follows a clear substitution logic: increasing the bio-oil fraction at fixed rubber content progressively decreases GWP, and increasing rubber from 20% to 30% further lowers the petroleum asphalt share but introduces more crumb-rubber uncertainty.

RA_20CR produced only a modest mean reduction relative to the control, despite replacing a substantial share of petroleum binder, because the rubber-processing burden and additional blending energy partly offset the avoided asphalt-binder production. BA_10BIO showed a larger and more stable mean reduction than RA_20CR because bio-oil displaces asphalt binder while contributing relatively low GWP in the base inventory. However, this result should not be generalized to all bio-oils. The specific bio-oil in this model is plant-based and represented by an energy inventory from Zhu et al. [13]; other bio-oils with high drying, upgrading, solvent, or refining burdens could produce different outcomes.

The exclusion of downstream stages may influence the overall sustainability conclusions. BAR binders may reduce pavement-level impacts if their rheological and mechanical performance extends service life or reduces maintenance frequency. Conversely, higher mixture-production energy, shortened durability, restricted recyclability, or unfavorable end-of-life treatment could offset or reverse the cradle-to-gate benefit. Accordingly, the present ranking should not be interpreted as a performance-equivalent pavement ranking without mixture-level and field-performance data.

### 3.2. Probability-Based Comparison with the Neat-Binder Control

**Figure 3:**
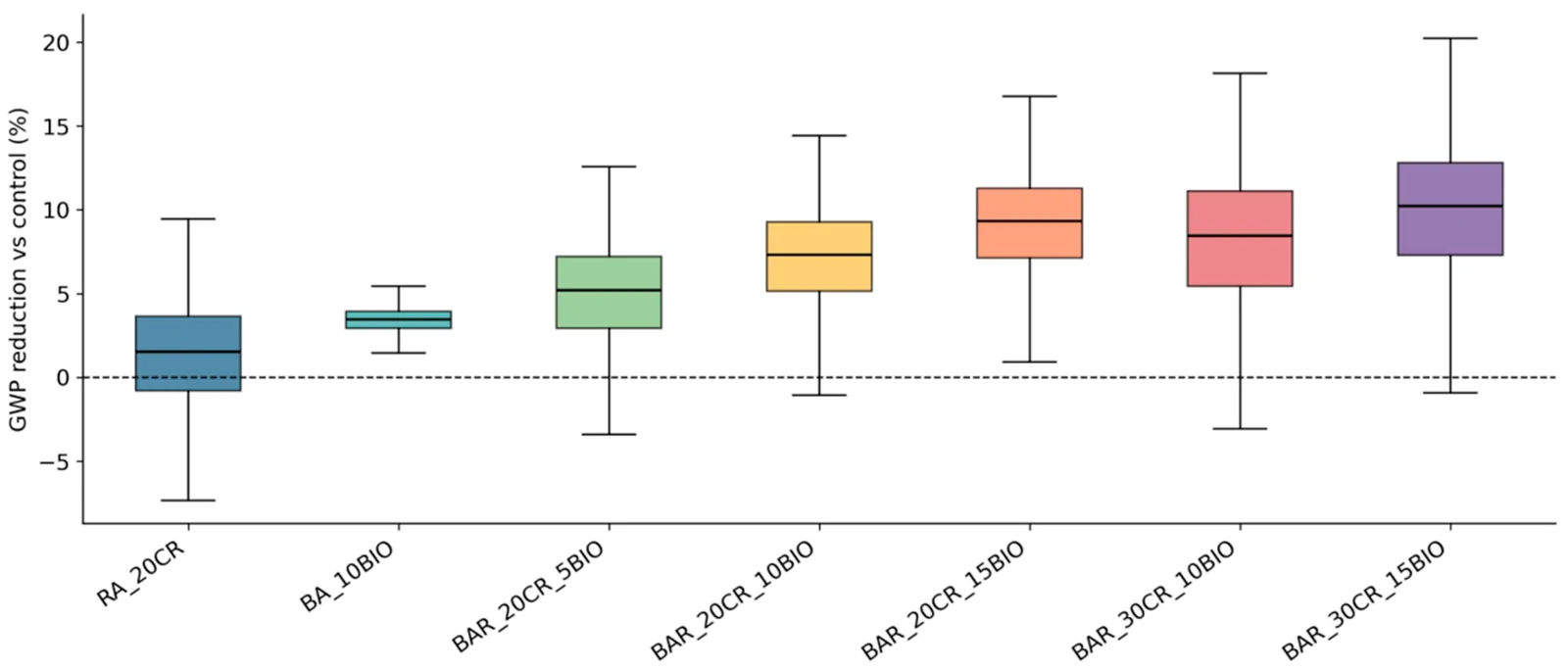
Distribution of GWP reduction relative to the neat-binder control under cut-off allocation. Positive values represent reductions relative to the control.

A probability-based interpretation changes the environmental ranking from a deterministic comparison into a risk statement, as shown in Figure 3 and Table 6. Under cut-off allocation, BA_10BIO had a 99.99% probability of lower GWP than the control, even though its mean reduction was only 3.4%. This indicates a small but highly robust binder-level advantage in the base inventory. RA_20CR had a 67.3% probability of lower GWP, showing that rubberized binder alone is not a statistically robust cradle-to-gate improvement when rubber is assigned only processing burdens and when additional blending energy is included.

**Figure 4:**
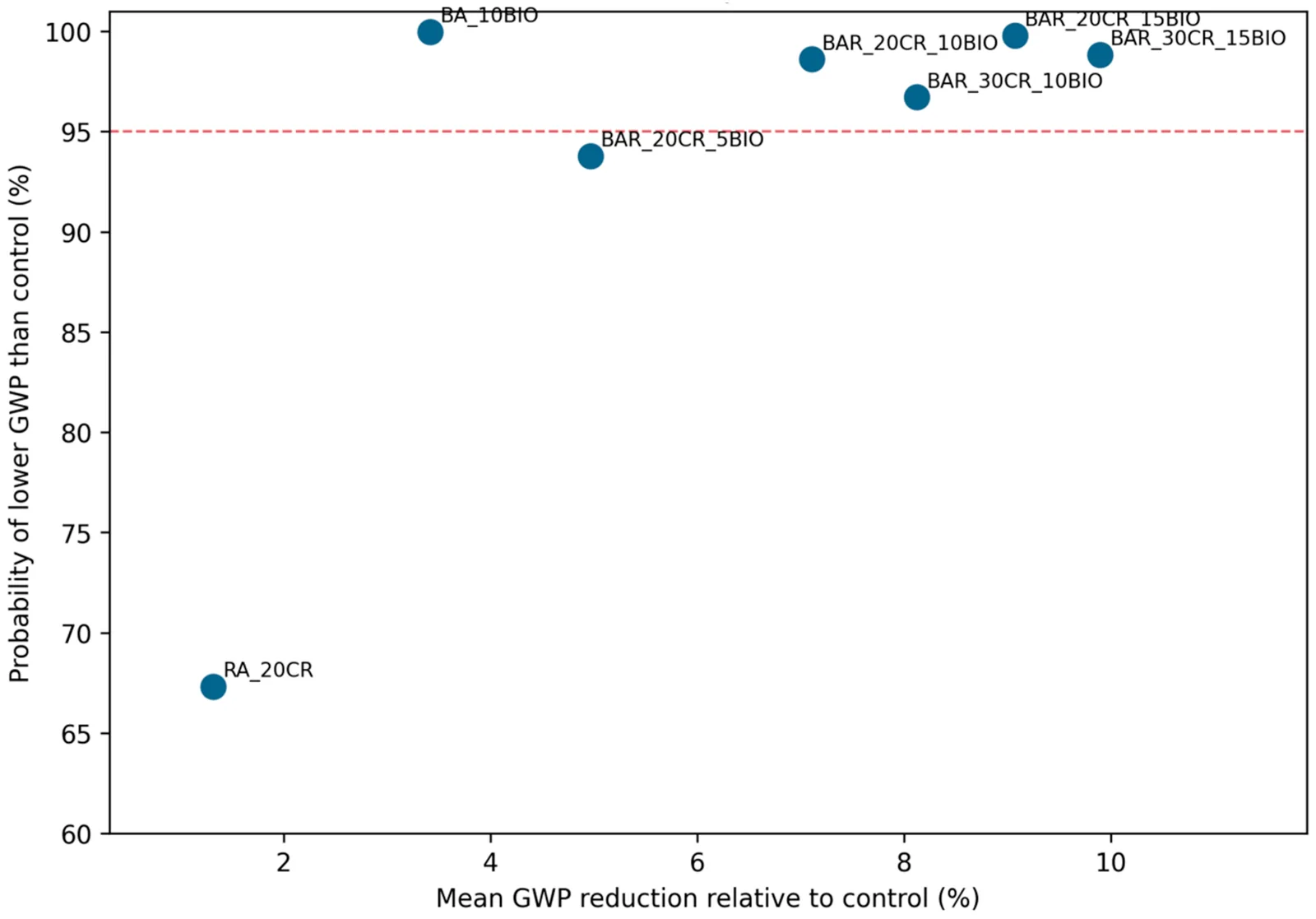
Environmental decision map under cut-off allocation. The horizontal dashed line marks a predefined decision threshold; alternatives above it have a high probability of outperforming the control.

BAR alternatives occupied an intermediate-to-strong preference region, as presented in Figure 4. BAR_20CR_5BIO had a 93.8% probability of improvement, slightly below a 95% preference threshold, because its lower bio-oil content leaves less petroleum-binder displacement while the rubber-processing and blending burdens remain. BAR_20CR_10BIO, BAR_20CR_15BIO, BAR_30CR_10BIO, and BAR_30CR_15BIO all exceeded 96% probability of outperforming the control. BAR_30CR_15BIO provided the largest mean reduction, 9.9%, and a 98.8% probability of lower GWP. These results suggest that BAR’s binder-level climate benefit is more robust when bio-oil and rubber are used together than when crumb rubber is used alone. The bio-oil component offsets part of the rubber-modification energy and processing burden by further reducing petroleum asphalt content.

### 3.3. Contribution Structure: Why Asphalt-Binder Substitution Dominates

**Figure 5:**
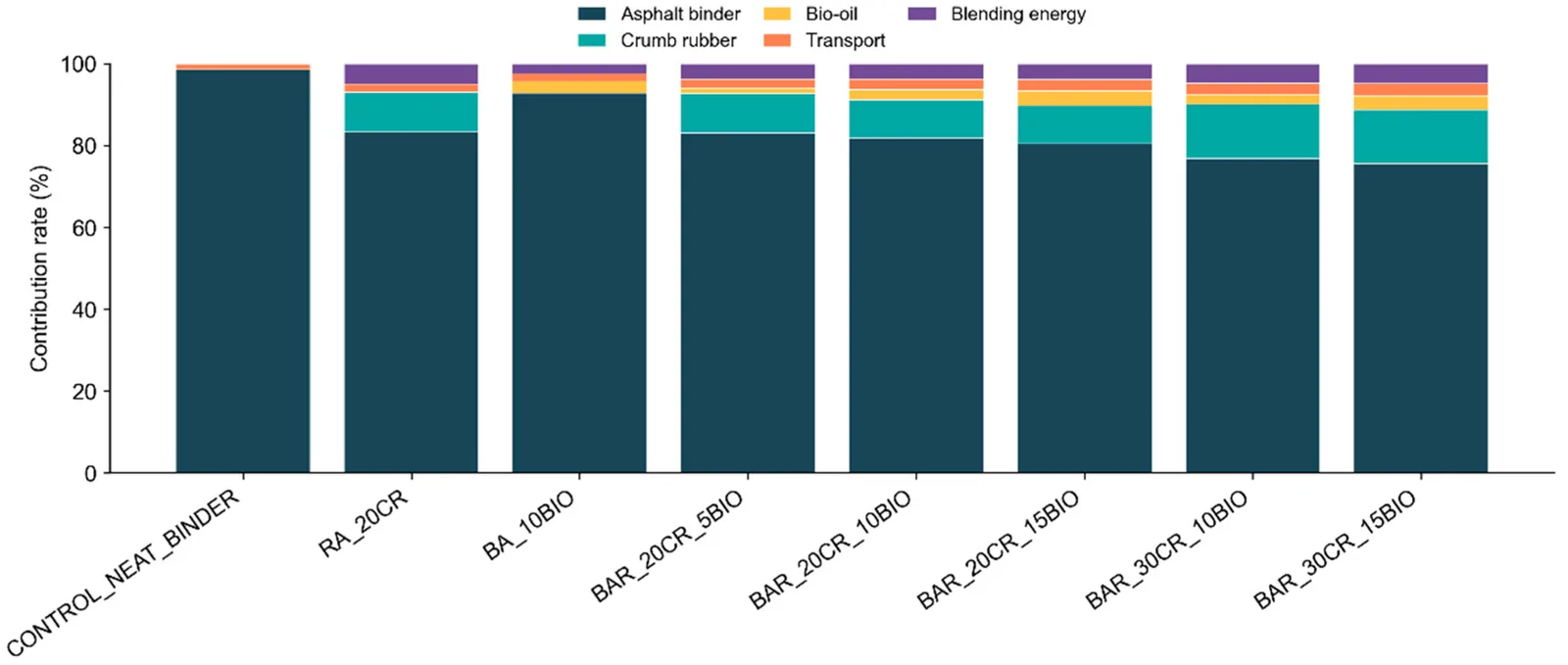
Mean cradle-to-gate GWP contribution rates under cut-off allocation.

The contribution analysis explains why the mean ranking is relatively stable under cut-off allocation, as shown in Table 7 and Figure 5. For the neat binder, asphalt-binder production accounted for 98.6% of total GWP, with inbound transport contributing only 1.4%. For BA_10BIO, asphalt binder still accounted for 92.8% of total GWP, while bio-oil production contributed only 2.8%. In the BAR alternatives, asphalt-binder production remained the largest source but declined from 83.1% in BAR_20CR_5BIO to 75.6% in BAR_30CR_15BIO. This shift shows that BAR reduces GWP primarily by diluting the high-GWP petroleum binder fraction rather than by eliminating process energy.

Crumb rubber contributed about 9.3–9.7% of total GWP in the 20% rubber BAR binders and about 13.2–13.4% in the 30% rubber BAR binders under cut-off allocation. Bio-oil contributed 1.3–3.7% depending on its dosage. Transport and production were comparatively small, but not negligible. Production energy contributed roughly 3.7–4.9% for BAR and RA binders, reflecting the extra blending energy needed for rubber-modified systems. These shares indicate that the environmental priority for BAR binder production should be: first, validate the asphalt-binder inventory and allocation method; second, obtain supplier-specific crumb-rubber processing data; third, improve bio-oil and blending-energy data.

### 3.4. Allocation Sensitivity: Cut-Off, Avoided Burden, and Clinker-Fuel System Expansion

**Figure 6:**
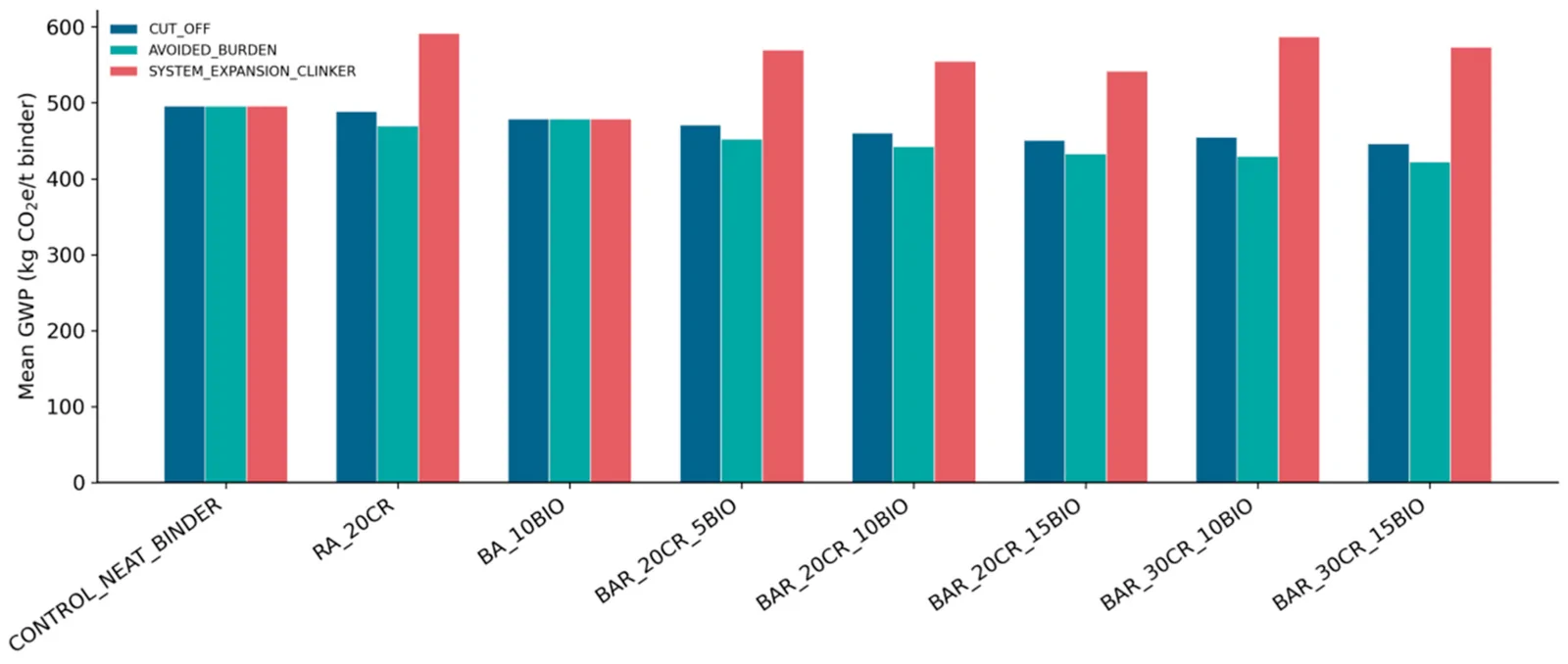
Crumb-rubber allocation sensitivity analysis.

The allocation sensitivity analysis is the most important interpretive result of the study, as presented in Figure 6 and Table 8. Under avoided-burden allocation, rubber-containing alternatives became substantially more favorable. Mean GWP declined to 452.1–422.0 kg CO2e/t for BAR alternatives, and the probability of lower GWP exceeded 99% for all BAR binders except BAR_20CR_5BIO, which still reached 99.3%. The largest mean reduction, 14.8%, occurred for BAR_30CR_15BIO. This scenario represents cases where BAR use prevents a low-value or environmentally burdensome tire-treatment pathway and credits the binder system for avoided management burdens.

In contrast, the system-expansion scenario against clinker-fuel substitution reversed the conclusion for all rubber-containing alternatives. RA_20CR increased to a mean GWP of 591.6 kg CO2e/t, and BAR alternatives increased to 541.7–587.0 kg CO2e/t. Their probabilities of outperforming the control fell to 0.3–5.1% for BAR and nearly zero for RA_20CR. BA_10BIO was unaffected by the rubber allocation scenario and remained below the control. This reversal does not mean that crumb rubber is environmentally undesirable in all cases; rather, it means that the environmental benefit of tire rubber depends on what the tire would have done otherwise. If regional markets already use tires efficiently as cement-kiln fuel with high avoided fossil-fuel benefits, diverting those tires to BAR has an opportunity cost. If tires would otherwise be landfilled, stockpiled, or downcycled, BAR may provide a strong benefit.

This result has direct implications for claims about circularity. A BAR binder is not automatically low-carbon because it contains waste rubber. Circularity claims should specify the counterfactual waste-management route and should report allocation sensitivity.

From an industrial implementation perspective, cut-off allocation is the most suitable default for supplier-level carbon accounting, procurement comparison, and EPD development because it provides a transparent and verifiable product-system boundary. Avoided-burden and system-expansion results are more relevant to regional policy and waste-management planning, where the displaced disposal or fuel-recovery pathway can be demonstrated. Accordingly, the cut-off results should be used as the primary product-level findings, while the other scenarios indicate the sensitivity of the conclusions to broader consequential assumptions.

### 3.5. Sensitivity of Total GWP to Inventory Parameters

**Figure 7:**
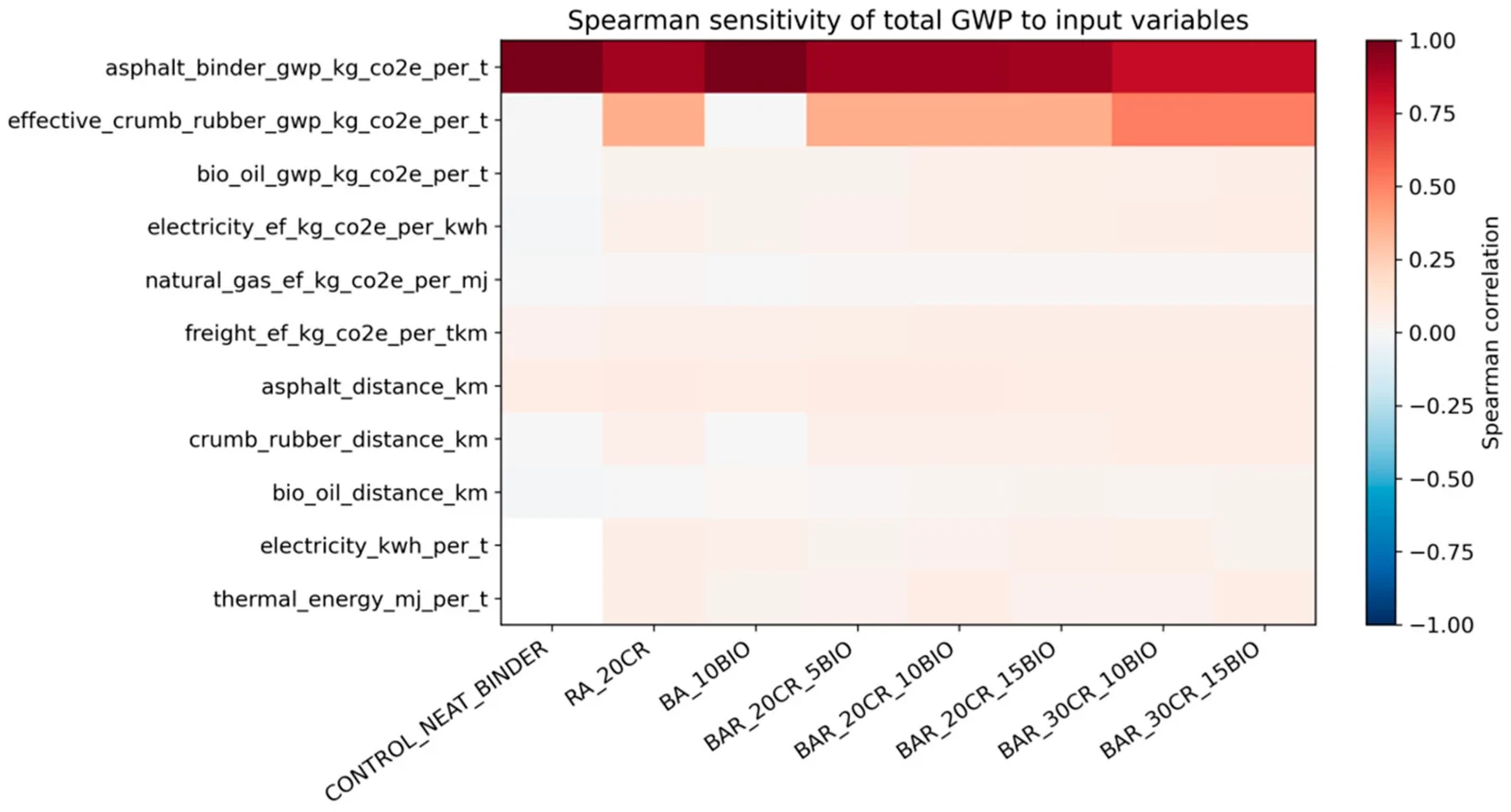
Spearman rank-correlation sensitivity of total GWP to uncertain input variables under cut-off allocation.

The sensitivity analysis (see Table 9 and Figure 7) confirms that asphalt-binder GWP is the dominant uncertainty for all alternatives, with Spearman correlations above 0.99 for the neat binder and BA_10BIO, around 0.90 for the 20% rubber systems, and around 0.82 for the 30% rubber BAR systems. As rubber content increases, the effective crumb-rubber GWP becomes increasingly influential, with correlations rising from approximately 0.37 in 20% rubber systems to approximately 0.51–0.52 in 30% rubber systems. This pattern mirrors the mass-balance structure: high-rubber BAR reduces asphalt-binder exposure but increases dependence on rubber-processing and allocation assumptions.

Bio-oil GWP had a smaller rank correlation than asphalt or crumb rubber, even in the high-bio-oil systems. This does not imply that bio-oil supply chains are unimportant; instead, it reflects the specific inventory adopted here, in which bio-oil production energy is low relative to asphalt-binder production on a per-ton basis and bio-oil makes up only 4–11% of the final binder mass. If a different bio-oil required high energy for drying, catalytic upgrading, solvent recovery, or long-distance transport, its sensitivity would increase. The model should therefore be used with supplier-specific bio-oil inventories for publication-quality case studies.

**Figure 8:**
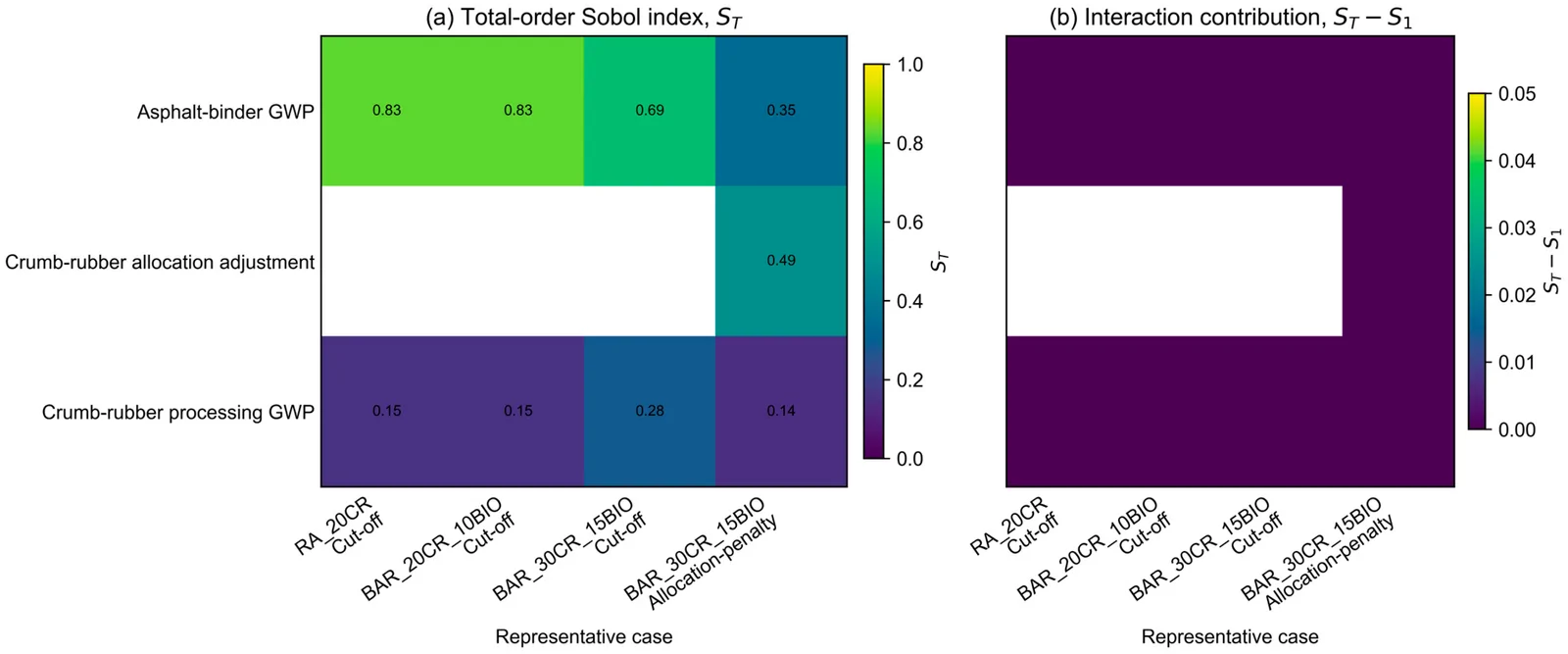
Variance-based global sensitivity of GWP.

Furthermore, the variance-based global sensitivity analysis (see Figure 8) generally confirmed the dominant role of the asphalt-binder inventory identified by the Spearman analysis, while providing a direct decomposition of output variance. Under cut-off allocation, asphalt-binder GWP had the highest total-order index for the evaluated formulations, with $S_{T}$ values of 0.829, 0.829, and 0.688for RA_20CR, BAR_20CR_10BIO, and BAR_30CR_15BIO, respectively. The influence of crumb-rubber processing increased with rubber content, reaching $S_{T}$ = 0.494 for BAR_30CR_15BIO. Other inventory, transportation, and blending-energy parameters individually contributed substantially less to total GWP uncertainty.

Under the allocation-penalty sensitivity, the crumb-rubber allocation adjustment became the dominant source of uncertainty, with $S_{T}$ = 0.494, followed by asphalt-binder GWP and crumb-rubber processing GWP. The differences between $S_{T}$ and $S_{1}$ were generally small, indicating that the model uncertainty was governed mainly by individual parameter effects. These results confirm that the environmental conclusion depends primarily on asphalt-binder inventory assumptions and, for high-rubber formulations, on crumb-rubber processing and allocation assumptions.

### 3.6. Interpretation for BAR Formulation Design

From a formulation perspective, the results identify three design principles. First, increasing bio-oil content from 5% to 15% at a fixed 20% crumb-rubber ratio lowered mean GWP from 470.8 to 450.4 kg CO2e/t under cut-off allocation. The improvement arises from greater petroleum-binder displacement and the relatively low GWP of the selected plant-based bio-oil. Second, increasing crumb rubber from 20% to 30% at a fixed 10% bio-oil ratio lowered mean GWP from 460.2 to 455.0 kg CO2e/t under cut-off allocation, but it also increased sensitivity to crumb-rubber allocation. Third, the best cut-off mean result, BAR_30CR_15BIO, also had the strongest exposure to system-expansion penalties. Thus, the binder with the lowest attributional GWP may not be the most robust option under consequential allocation.

A risk-aware formulation strategy would therefore avoid optimizing only the mean GWP under one allocation rule. If the target is a conservative attributional EPD, BAR_30CR_15BIO appears attractive because it has the lowest mean GWP and a high probability of outperforming the neat binder within the model and inventory adopted in this study. If the target is robust performance across allocation assumptions, BA_10BIO is more allocation-insensitive because it does not depend on tire-rubber credits. If the target is high-value tire circularity under a cut-off policy, BAR_20CR_10BIO or BAR_20CR_15BIO may be more balanced because they offer a high probability of lower GWP while reducing the risk associated with very high rubber content.

## 4. Conclusions

This study investigates the environmental impact of BAR binders using the probabilistic LCA approach, and the primary conclusions can be drawn as follows:

(1) A probabilistic cradle-to-gate LCA framework was developed for bio-asphalt–rubber binders using one metric ton of final binder at plant gate as the declared unit. The framework combines mass-normalized BAR formulations, the literature-based LCI ranges, a specific plant-based bio-oil energy inventory, and crumb-rubber allocation sensitivity.
(2) Under cut-off crumb-rubber allocation, all BAR alternatives had lower mean GWP than the neat petroleum binder within the modeled inventory. Mean GWP decreased from 495.9 kg CO2e/t for CONTROL_NEAT_BINDER to 470.8, 460.2, 450.4, 455.0, and 446.2 kg CO2e/t for the five BAR alternatives. BAR_30CR_15BIO had the lowest mean impact and a 98.8% probability of lower GWP than the control.
(3) The probability-based results were more informative than deterministic means. RA_20CR reduced mean GWP by only 1.3% and had a 67.3% probability of outperforming the control, whereas BAR alternatives with at least 10% bio-oil showed probabilities above 96%. Bio-oil addition therefore improves the robustness of the cradle-to-gate benefit in the modeled inventory for the selected bio-oil pathway, regional electricity and transport conditions, and end-of-life tire counterfactual.
(4) Contribution analysis showed that petroleum asphalt binder remains the dominant GWP contributor, accounting for 75.6–83.1% of total GWP in BAR alternatives under cut-off allocation. Crumb rubber contributed 9.3–13.4%, bio-oil 1.3–3.7%, transport 2.3–2.9%, and blending energy 3.7–4.9%.
(5) Crumb-rubber allocation controlled the environmental conclusion. Avoided-burden allocation strengthened BAR’s advantage, with mean reductions up to 14.8%. System expansion against clinker-fuel substitution reversed the conclusion for rubber-containing alternatives, reducing their probability of outperforming the control to below 6%.
(6) BAR binders can provide a cradle-to-gate climate benefit, but this benefit is not an intrinsic material property. It is conditional on the asphalt-binder inventory, the bio-oil supply chain, the regional end-of-life tire counterfactual, and the chosen allocation method. Future pavement-level analyses should incorporate mixture production, field performance, maintenance, use-stage emissions, and end-of-life recovery.

Finally, some limitations in this study should be acknowledged. Because the bio-oil inventory represents a single documented production pathway, the reported values should be interpreted as generic screening results. The relative advantage of BAR binders may change with bio-oil feedstock and conversion technology. In addition, the triangular distributions represent bounded knowledge uncertainty rather than empirically fitted variability. Although this approach is transparent under data scarcity, the resulting uncertainty intervals may remain sensitive to the assumed distribution form. Also, this study is limited to a declared unit of one metric ton of binder and a cradle-to-gate boundary; other stages outside this boundary may strengthen, reduce, or reverse the reported findings. Integrating pavement performance models with life-cycle assessment would significantly improve the practical impact of the study.

## Figures and Tables

**Table 1:** System boundary adopted for the binder-level cradle-to-gate assessment.

Module	Included Unit Processes	Excluded Processes
A1	Petroleum asphalt binder production; crumb-rubber processing; plant-based bio-oil production	Aggregate production, asphalt-mixture production, field placement, service life effects
A2	Inbound freight transport for asphalt binder, crumb rubber, and bio-oil to binder-blending facility	Transport to asphalt-mix plant and paving site
A3	Binder blending electricity and thermal energy	Mixture heating, silo storage, construction equipment
Beyond gate	None	Use phase, M&R, end-of-life, RAP recovery, long-term biogenic carbon release or storage

**Table 2:** Binder alternatives and mass-normalized composition for one metric ton of final binder.

Alternative	CR Ratio to Neat Asphalt	Bio-Oil Ratio to Neat Asphalt	Asphalt in Final Binder (t/t)	CR in Final Binder (t/t)	Bio-Oil in Final Binder (t/t)	A3 Blending Basis
CONTROL_NEAT_BINDER	0.000	0.000	1.000	0.000	0.000	None
RA_20CR	0.200	0.000	0.833	0.167	0.000	177 °C, 60 min
BA_10BIO	0.000	0.100	0.909	0.000	0.091	160 °C, 30 min
BAR_20CR_5BIO	0.200	0.050	0.800	0.160	0.040	160 °C, 30 min
BAR_20CR_10BIO	0.200	0.100	0.769	0.154	0.077	160 °C, 30 min
BAR_20CR_15BIO	0.200	0.150	0.741	0.148	0.111	160 °C, 30 min
BAR_30CR_10BIO	0.300	0.100	0.714	0.214	0.071	160 °C, 30 min
BAR_30CR_15BIO	0.300	0.150	0.690	0.207	0.103	160 °C, 30 min

**Table 3:** Cradle-to-gate LCI parameters and uncertainty ranges used in the Monte Carlo model.

Parameter	Module	Distribution (Low/Mode/High)
asphalt_binder_gwp_kg_co2e_per_t	A1	triangular: 390.0/500.0/578.0 kg CO2e/t
crumb_rubber_processing_gwp_kg_co2e_per_t	A1	triangular: 100.0/250.0/500.0 kg CO2e/t
bio_oil_production_liquid_fuel_l_per_t	A1	triangular: 37.0/41.3/46.0 L/t
bio_oil_production_electricity_kwh_per_t	A1	triangular: 88.0/97.8/108.0 kWh/t
liquid_fuel_ef_kg_co2e_per_l	A1/A3	triangular: 2.49/2.68/2.95 kg CO2e/L
electricity_ef_kg_co2e_per_kwh	A1/A3	triangular: 0.2/0.37/0.55 kg CO2e/kWh
natural_gas_ef_kg_co2e_per_mj	A3	triangular: 0.05/0.053/0.06 kg CO2e/MJ
freight_ef_kg_co2e_per_tkm	A2	triangular: 0.045/0.062/0.095 kg CO2e/t-km
asphalt_distance_km	A2	triangular: 30.0/80.0/200.0 km
crumb_rubber_distance_km	A2	triangular: 80.0/250.0/800.0 km
bio_oil_distance_km	A2	triangular: 80.0/230.0/1000.0 km

Note: asphalt_binder_gwp_kg_co2e_per_t denotes the cradle-to-gate greenhouse gas emission factor of virgin petroleum asphalt binder, expressed as kg CO_2_-eq per metric ton of binder. crumb_rubber_processing_gwp_kg_co2e_per_t represents the processing-related emission factor of crumb rubber, including waste tire collection, shredding, grinding, separation, and sizing, expressed as kg CO_2_-eq per metric ton of crumb rubber. bio_oil_production_liquid_fuel_l_per_t and bio_oil_production_electricity_kwh_per_t describe the liquid fuel and electricity requirements for producing one metric ton of bio-oil, respectively. liquid_fuel_ef_kg_co2e_per_l, electricity_ef_kg_co2e_per_kwh, and natural_gas_ef_kg_co2e_per_mj are the emission factors for liquid fuel combustion, electricity consumption, and natural gas use, respectively. freight_ef_kg_co2e_per_tkm denotes the freight transport emission factor per ton-kilometer. asphalt_distance_km, crumb_rubber_distance_km, and bio_oil_distance_km represent the assumed one-way transport distances from the asphalt binder supplier, crumb rubber producer, and bio-oil producer to the binder blending facility, respectively.

**Table 4:** Crumb-rubber allocation scenarios and adjustment ranges applied to the cut-off processing burden.

Scenario	Description	Low Adjustment	Mode Adjustment	High Adjustment
Cut off	Only crumb-rubber collection/grinding/processing burdens are assigned to crumb rubber; no credit or penalty for avoided alternative tire treatment.	0	0	0
Avoided burden	Sensitivity case where use of crumb rubber in BAR is credited for avoided landfill/MSWI-type treatment and related management burdens.	−250	−100	0
System expansion	Sensitivity case where waste tires would otherwise be used as fuel in clinker production; diverting them to BAR creates a foregone-fuel-substitution burden.	250	600	1000

**Table 5:** Probabilistic cradle-to-gate GWP summary under cut-off crumb-rubber allocation (kg CO2e/t binder).

Alternative	Mean	Std. Dev.	P05	P25	Median	P75	P95
CONTROL_NEAT_BINDER	495.9	38.67	428.4	468.2	498.3	523.9	558.8
RA_20CR	488.9	35.32	428.7	464.5	489.8	514.1	546.4
BA_10BIO	478.8	35.29	417.6	453.4	480.9	504.0	535.8
BAR_20CR_5BIO	470.8	33.82	413.4	447.2	471.7	494.6	525.8
BAR_20CR_10BIO	460.2	32.64	404.7	437.6	460.6	483.2	513.5
BAR_20CR_15BIO	450.4	31.43	396.9	428.8	451.1	472.9	501.4
BAR_30CR_10BIO	455.0	33.15	398.7	431.9	455.3	478.4	509.5
BAR_30CR_15BIO	446.2	32.08	392.1	424.0	446.6	468.5	499.0

**Table 6:** Probability of lower GWP and reduction distribution relative to the neat-binder control under cut-off allocation.

Alternative	Probability Lower Than Control (%)	Mean Reduction (%)	Median Reduction (%)	P05 Reduction (%)	P95 Reduction (%)
CONTROL_NEAT_BINDER	0.000	0.000	0.000	0.000	0.000
RA_20CR	67.32	1.317	1.537	−4.314	6.162
BA_10BIO	99.99	3.417	3.471	2.132	4.546
BAR_20CR_5BIO	93.78	4.970	5.205	−0.433	9.562
BAR_20CR_10BIO	98.61	7.109	7.321	1.828	11.64
BAR_20CR_15BIO	99.80	9.072	9.314	3.808	13.57
BAR_30CR_10BIO	96.72	8.126	8.455	0.864	14.23
BAR_30CR_15BIO	98.84	9.896	10.23	2.844	15.92

**Table 7:** Mean GWP contribution by component under cut-off allocation (kg CO2e/t binder).

Alternative	Asphalt GWP	CR GWP	Bio-Oil GWP	Transport GWP	Production GWP	Total GWP
CONTROL_NEAT_BINDER	489.0	0.000	0.000	6.949	0.000	495.9
RA_20CR	407.5	47.35	0.000	10.03	24.06	488.9
BA_10BIO	444.5	0.000	13.52	9.008	11.75	478.8
BAR_20CR_5BIO	391.2	45.46	5.949	10.81	17.41	470.8
BAR_20CR_10BIO	376.1	43.71	11.44	11.53	17.36	460.2
BAR_20CR_15BIO	362.2	42.09	16.53	12.20	17.39	450.4
BAR_30CR_10BIO	349.3	60.88	10.62	12.53	21.69	455.0
BAR_30CR_15BIO	337.2	58.78	15.39	13.12	21.69	446.2

**Table 8:** Probability and reduction sensitivity across crumb-rubber allocation scenarios.

Allocation Scenario	Alternative	Probability Lower Than Control (%)	Mean Reduction (%)	Median Reduction (%)	P05 Reduction (%)	P95 Reduction (%)
Cut off	CONTROL_NEAT_BINDER	0.000	0.000	−0.000	−0.000	0.000
Cut off	RA_20CR	67.32	1.317	1.537	−4.314	6.162
Cut off	BA_10BIO	99.99	3.417	3.471	2.132	4.546
Cut off	BAR_20CR_5BIO	93.78	4.970	5.205	−0.433	9.562
Cut off	BAR_20CR_10BIO	98.61	7.109	7.321	1.828	11.64
Cut off	BAR_20CR_15BIO	99.80	9.072	9.314	3.808	13.57
Cut off	BAR_30CR_10BIO	96.72	8.126	8.455	0.864	14.23
Cut off	BAR_30CR_15BIO	98.84	9.896	10.23	2.844	15.92
Avoided burden	CONTROL_NEAT_BINDER	0.000	0.000	0.000	0.000	0.000
Avoided burden	RA_20CR	92.67	5.275	5.396	−0.729	10.92
Avoided burden	BA_10BIO	99.99	3.417	3.471	2.132	4.546
Avoided burden	BAR_20CR_5BIO	99.31	8.770	8.904	3.015	14.10
Avoided burden	BAR_20CR_10BIO	99.91	10.76	10.89	5.095	15.96
Avoided burden	BAR_20CR_15BIO	100.0	12.59	12.74	6.971	17.71
Avoided burden	BAR_30CR_10BIO	99.70	13.21	13.37	5.509	20.38
Avoided burden	BAR_30CR_15BIO	99.94	14.81	14.95	7.227	21.76
System expansion	CONTROL_NEAT_BINDER	0.000	0.000	0.000	0.000	0.000
System expansion	RA_20CR	0.010	−19.52	−19.17	−30.87	−9.041
System expansion	BA_10BIO	99.99	3.417	3.471	2.132	4.546
System expansion	BAR_20CR_5BIO	0.330	−15.03	−14.69	−25.94	−4.979
System expansion	BAR_20CR_10BIO	1.540	−12.12	−11.79	−22.74	−2.411
System expansion	BAR_20CR_15BIO	5.120	−9.449	−9.137	−19.85	0.081
System expansion	BAR_30CR_10BIO	0.660	−18.66	−18.19	−33.36	−5.145
System expansion	BAR_30CR_15BIO	1.780	−15.97	−15.53	−30.12	−2.879

**Table 9:** Top three Spearman sensitivity parameters for each alternative under cut-off allocation.

Alternative	Ranked Sensitive Input	Spearman Rho
BAR_20CR_10BIO	asphalt_binder_gwp_kg_co2e_per_t	0.906
BAR_20CR_10BIO	effective_crumb_rubber_gwp_kg_co2e_per_t	0.372
BAR_20CR_10BIO	asphalt_distance_km	0.079
BAR_20CR_15BIO	asphalt_binder_gwp_kg_co2e_per_t	0.904
BAR_20CR_15BIO	effective_crumb_rubber_gwp_kg_co2e_per_t	0.372
BAR_20CR_15BIO	asphalt_distance_km	0.078
BAR_20CR_5BIO	asphalt_binder_gwp_kg_co2e_per_t	0.908
BAR_20CR_5BIO	effective_crumb_rubber_gwp_kg_co2e_per_t	0.372
BAR_20CR_5BIO	asphalt_distance_km	0.080
BAR_30CR_10BIO	asphalt_binder_gwp_kg_co2e_per_t	0.822
BAR_30CR_10BIO	effective_crumb_rubber_gwp_kg_co2e_per_t	0.517
BAR_30CR_10BIO	asphalt_distance_km	0.076
BAR_30CR_15BIO	asphalt_binder_gwp_kg_co2e_per_t	0.821
BAR_30CR_15BIO	effective_crumb_rubber_gwp_kg_co2e_per_t	0.514
BAR_30CR_15BIO	asphalt_distance_km	0.075
BA_10BIO	asphalt_binder_gwp_kg_co2e_per_t	0.994
BA_10BIO	asphalt_distance_km	0.075
BA_10BIO	freight_ef_kg_co2e_per_tkm	0.053
CONTROL_NEAT_BINDER	asphalt_binder_gwp_kg_co2e_per_t	0.998
CONTROL_NEAT_BINDER	asphalt_distance_km	0.075
CONTROL_NEAT_BINDER	freight_ef_kg_co2e_per_tkm	0.042
RA_20CR	asphalt_binder_gwp_kg_co2e_per_t	0.906
RA_20CR	effective_crumb_rubber_gwp_kg_co2e_per_t	0.372
RA_20CR	asphalt_distance_km	0.078

## Data Availability

The original contributions presented in the study are included in the article, further inquiries can be directed to the corresponding author.
